# Denitrification Aligns with N_2_ Fixation in Red Sea Corals

**DOI:** 10.1038/s41598-019-55408-z

**Published:** 2019-12-19

**Authors:** Arjen Tilstra, Yusuf C. El-Khaled, Florian Roth, Nils Rädecker, Claudia Pogoreutz, Christian R. Voolstra, Christian Wild

**Affiliations:** 1 Marine Ecology Department, Faculty of Biology and Chemistry, University of Bremen, Bremen, 28359 Germany; 2 Red Sea Research Center, King Abdullah University of Science and Technology, Thuwal, 23955-6900 Kingdom of Saudi Arabia; 3 Department of Biology, University of Konstanz, Konstanz, 78464 Germany

**Keywords:** Molecular ecology, Ecophysiology

## Abstract

Denitrification may potentially alleviate excess nitrogen (N) availability in coral holobionts to maintain a favourable N to phosphorous ratio in the coral tissue. However, little is known about the abundance and activity of denitrifiers in the coral holobiont. The present study used the *nirS* marker gene as a proxy for denitrification potential along with measurements of denitrification rates in a comparative coral taxonomic framework from the Red Sea: *Acropora hemprichii*, *Millepora dichotoma*, and *Pleuractis granulosa*. Relative *nirS* gene copy numbers associated with the tissues of these common corals were assessed and compared with denitrification rates on the holobiont level. In addition, dinitrogen (N_2_) fixation rates, Symbiodiniaceae cell density, and oxygen evolution were assessed to provide an environmental context for denitrification. We found that relative abundances of the *nirS* gene were 16- and 17-fold higher in *A. hemprichii* compared to *M. dichotoma* and *P. granulosa*, respectively. In concordance, highest denitrification rates were measured in *A. hemprichii*, followed by *M. dichotoma* and *P. granulosa*. Denitrification rates were positively correlated with N_2_ fixation rates and Symbiodiniaceae cell densities. Our results suggest that denitrification may counterbalance the N input from N_2_ fixation in the coral holobiont, and we hypothesize that these processes may be limited by photosynthates released by the Symbiodiniaceae.

## Introduction

Corals are holobionts consisting of the coral host and a diverse microbiome composed of Symbiodiniaceae (i.e., endosymbiotic dinoflagellates capable of photosynthesis), and prokaryotes, i.e. bacteria and archaea, among other microbes^1^. Complex symbiotic interactions within these holobionts render corals mixotrophic, that is they can obtain nutrients through both autotrophic and heterotrophic means^2–4^. The endosymbiotic dinoflagellates, belonging to the family Symbiodiniaceae^5^, provide the coral with a substantial part of their metabolic energy via autotrophy in the form of photosynthetically fixed carbon (C)^6^. In return, the Symbiodiniaceae require nutrients from the coral host, e.g. nitrogen (N) and phosphorous (P), which can be obtained via heterotrophic feeding or by uptake from the water column and/or internal (re)cycling^7,8^.

The involvement of prokaryotes in holobiont nutrient cycling has received increasing attention in recent years. Diazotrophs in particular (microbes capable of fixing atmospheric dinitrogen (N_2_)) ubiquitously occur in corals^9–11^ and are recognized as an important source of N for holobiont productivity^12–16^. Diazotrophs can provide the holobiont with bioavailable N in the form of ammonium, a preferred N source for Symbiodiniaceae^17–19^, in particular in times of N scarcity^11^.

**Figure 1:**
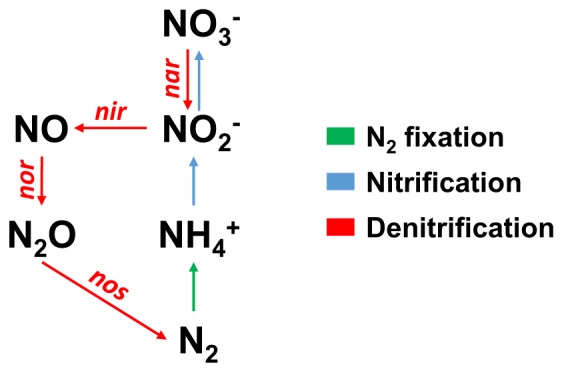
Schematic representation of three major pathways involved in nitrogen cycling, including the four gene clusters responsible for denitrification. N_2_ = atmospheric nitrogen, NH_4_^+^ = ammonium, NO_2_^−^ = nitrite, NO_3_^−^ = nitrate, NO = nitric oxide, N_2_O = nitrous oxide, *nar* = gene cluster for nitrate reductase, *nir* = gene cluster for nitrite reductase, *nor* = gene cluster for nitric oxide reductase, *nos* = gene cluster for nitrous oxide reductase.

Excess (microbial) input of bioavailable N into the coral holobiont can potentially lead to a misbalance of the N:P ratio, i.e. a shift from N towards P limitation, thereby increasing bleaching susceptibility^14,20,21^. Previously, it was hypothesized that the activity of other N-cycling microbes could alleviate coral holobionts from nutrient stress via the removal of nitrogenous compounds^22^. Indeed, ammonium oxidizing (i.e. nitrifying) and nitrate reducing (i.e. denitrifying) prokaryotes occur ubiquitously on coral reefs^23–27^, including coral holobionts^22,28,29^. The denitrification pathway in particular may be important for holobiont functioning as it effectively removes bioavailable N. Here, nitrate is reduced to atmospheric N_2_ via the activity of four main enzymes, i.e. nitrate reductase (converting nitrate to nitrite), nitrite reductase (converting nitrite to nitric oxide), nitric oxide reductase (converting nitric oxide to nitrous oxide), and nitrous oxide reductase (converting nitrous oxide to N_2_) (Fig. 1)^30,31^. While Symbiodiniaceae cells are considered a major N sink in the coral holobiont^32^, excess N could potentially be removed by denitrifying microbes to help maintain an N-limited state^14^. However, whether removal of excess N via denitrification contributes to holobiont functioning and health remains poorly understood.

Recently, Pogoreutz *et al*.^10^ demonstrated that N_2_ fixation rates may not only be species-specific, but align with relative gene copy numbers and expression of the *nifH* gene. This pattern was linked to heterotrophic capacity of the investigated corals. However, it is unknown how these patterns of N_2_ fixation activity ultimately relate to other N-cycling processes, i.e. denitrification, within the coral holobiont. The present study thus aimed to answer (*i*) whether patterns of denitrification are coral species-specific; (*ii*) whether relative abundances of the *nirS* gene (denitrification potential) can be related to denitrification rates; and (*iii*) whether denitrification aligns with other biological variables within the coral holobiont, specifically N_2_ fixation, photosynthesis, and cell density of Symbiodiniaceae. These questions were answered in a comparative taxonomic framework of three common Red Sea coral species. Relative gene copy numbers of the *nirS* gene, which encodes for a nitrite reductase containing cytochrome *cd*_1_, were assessed by qPCR to serve as a proxy for denitrification potential of coral tissue-associated prokaryotes. Relative quantification of *nirS* gene copy numbers was achieved by referencing against the ITS2 region of Symbiodiniaceae. Denitrification and N_2_ fixation rates were quantified indirectly using a COmbined Blockage/Reduction Acetylene (COBRA) assay (El-Khaled *et al*. unpublished). Finally, Symbiodiniaceae cell densities were manually counted and photosynthesis was assessed by measuring O_2_ fluxes.

## Results

### Relative abundances of the *nirS* gene and denitrification rates

**Figure 2:**
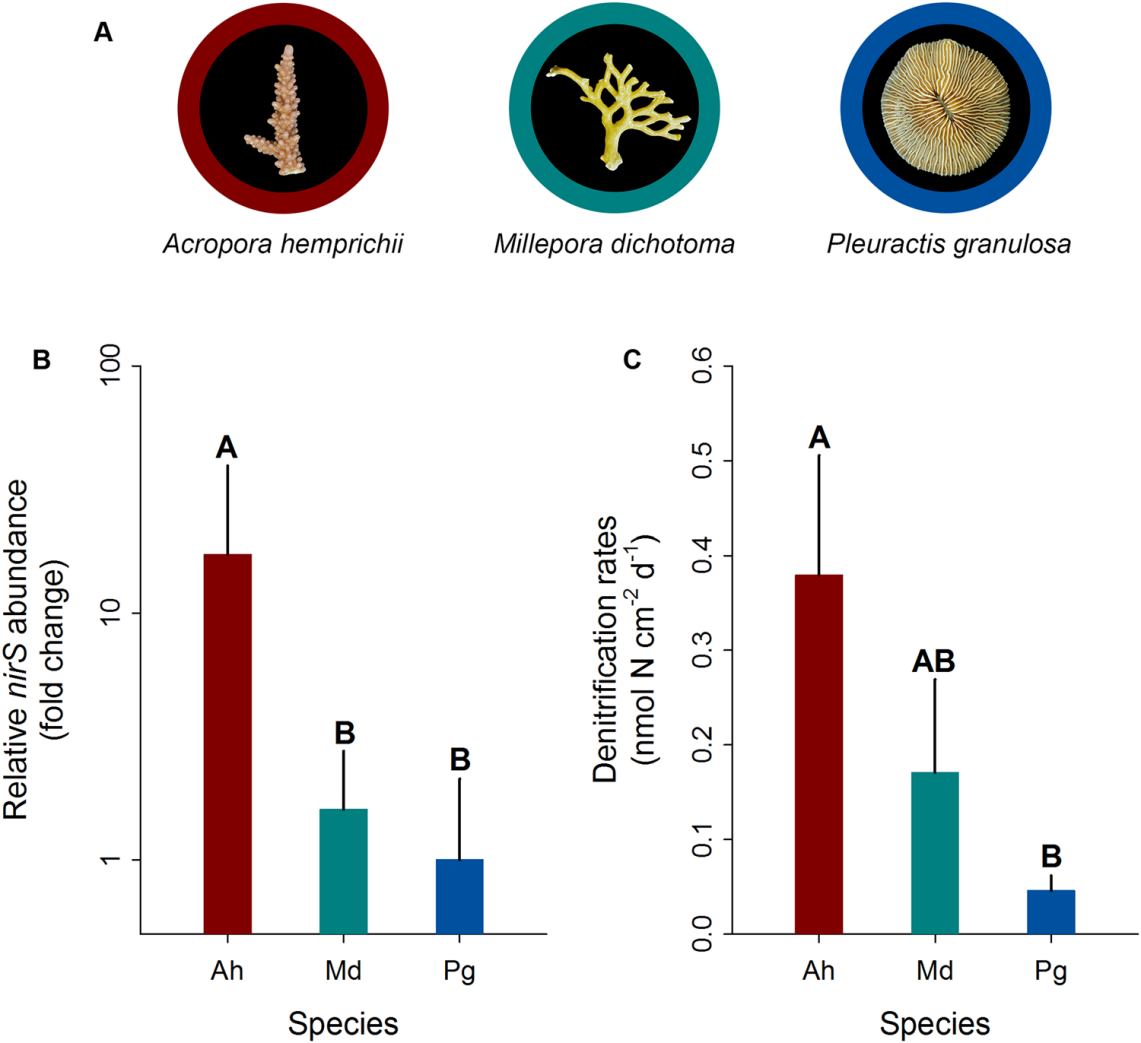
Relative *nirS* gene copy numbers and rates of denitrification associated with three Red Sea coral species. (**A**) Representative photographs of investigated species, (**B**) fold changes in relative *nirS* gene copy numbers normalized to ITS2 copy numbers as measured by quantitative PCR, and (**C**) denitrification rates measured indirectly via the combined blockage/reduction acetylene assay (COBRA-assay). Ah = *A. hemprichii*, Md = *M. dichotoma*, and Pg = *P. granulosa*. Fold changes were calculated in relation to *P. granulosa*; bars indicate the mean; error bars indicate upper confidence intervals (+ 1 SE); n = 4 per species, except Ah and Pg in (**B**) (n = 3). Different letters above error bars indicate statistically significant differences between groups within each figure (pair-wise PERMANOVAs, *p* < 0.05).

The qPCR confirmed the presence of the *nirS* gene in the tissues of all investigated corals (Fig. 2A,B). *Acropora hemprichii* exhibited significantly higher relative *nirS* gene copy numbers compared to *M. dichotoma* (~16-fold; pair-wise PERMANOVA, t = 3.82, *p* = 0.015) and *P. granulosa* (~17-fold; pair-wise PERMANOVA, t = 3.25, *p* = 0.029). A similar pattern was found for denitrification rates (Fig. 2C). *Acropora hemprichii* exhibited the highest denitrification rates (~0.38 ± 0.13 nmol N cm^−2^ d^−1^), followed by *M. dichotoma* (~0.17 ± 0.10 nmol N cm^−2^ d^−1^) and *P. granulosa* (~0.05 ± 0.02 nmol N cm^−2^ d^−1^). Denitrification rates in *A. hemprichii* were significantly different from those in *P. granulosa* (pair-wise PERMANOVA, t = 2.75, *p* = 0.036), but not those measured in *M. dichotoma* (pair-wise PERMANOVA, t = 1.31, *p* = 0.237). Finally, denitrification rates in *M. dichotoma* were not significantly different from those in *P. granulosa* (pair-wise PERMANOVA, t = 1.23, *p* = 0.264).

### N_2_ fixation, Symbiodiniaceae cell density and O_2_ fluxes

**Figure 3:**
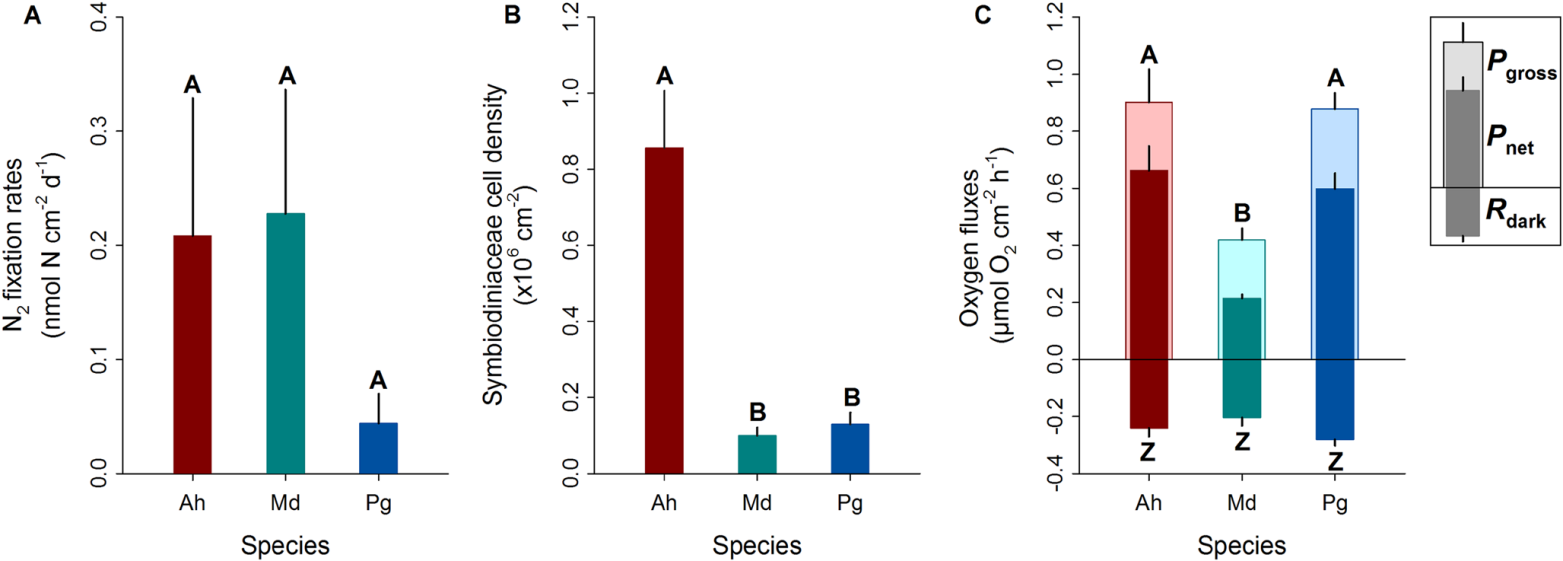
Biological variables of three Red Sea corals. (**A**) N_2_ fixation rates measured indirectly using a COBRA assay, (**B**) Symbiodiniaceae cell densities, and (**C**) oxygen fluxes. Ah = *Acropora hemprichii*, Md = *Millepora dichotoma*, and Pg = *Pleuractis granulosa*. *P*_gross_ = gross photosynthesis, *P*_net_ = net photosynthesis, *R*_dark_ = dark respiration. Bars indicate the mean; error bars indicate upper confidence intervals (+1 SE); n = 4 per species. Different letters above error bars indicate statistically significant differences within each plot (pair-wise PERMANOVAs, *p* < 0.05); differences in (**C**) apply to both *P*_gross_ and *P*_net_.

N_2_ fixation rates were highest in *M. dichotoma* (0.23 ± 0.11 nmol N cm^−2^ d^−1^), followed by *A. hemprichii* (0.21 ± 0.12 nmol N cm^−2^ d^−1^), and lowest in *P. granulosa* (0.04 ± 0.03 nmol N cm^−2^ d^−1^) (Fig. 3A). Due to high biological variation in the samples, these differences between coral species were not significant (Fig. 3A).

Cell densities of Symbiodiniaceae were significantly higher in *A. hemprichii* (0.86 ± 0.15 × 10^6^ cells cm^−2^) compared to *M. dichotoma* (0.10 ± 0.02 × 10^6^ cells cm^−2^; pair-wise PERMANOVA, t = 6.86, *p* < 0.001) and *P. granulosa* (0.13 ± 0.03 × 10^6^ cells cm^−2^; pair-wise PERMANOVA, t = 5.03, *p* = 0.003) (Fig. 3B). Cell densities of Symbiodiniaceae in *M. dichotoma* and *P. granulosa* did not differ significantly (Fig. 3B).

Significantly lower *P*_net_ was found for *M. dichotoma* (0.21 ± 0.01 µmol O_2_ cm^−2^ h^−1^) compared to that of *A. hemprichii* (0.66 ± 0.09 µmol O_2_ cm^−2^ h^−1^; pair-wise PERMANOVA, t = 6.55, *p* = 0.002) and that of *P. granulosa* (0.60 ± 0.05 µmol O_2_ cm^−2^ h^−1^; pair-wise = PERMANOVA, t = 8.95, *p* < 0.001) (Fig. 3C). The same pattern was found for *P*_gross_; *M. dichotoma* exhibited significantly lower *P*_gross_ (0.42 ± 0.04 µmol O_2_ cm^−2^ h^−1^) than that of *A. hemprichii* (0.90 ± 0.12 µmol O_2_ cm^−2^ h^−1^; pair-wise PERMANOVA, t = 4.14, *p* = 0.007) and that of *P. granulosa* (0.88 ± 0.06 µmol O_2_ cm^−2^ h^−1^; pair-wise PERMANOVA, t = 6.21, *p* = 0.002) (Fig. 3C). No significant differences were found for *R*_dark_ between species (PERMANOVA, Pseudo-F = 1.73, p = 0.243) (Fig. 3C).

### Comparison of denitrification rates and N_2_ fixation rates

**Figure 4:**
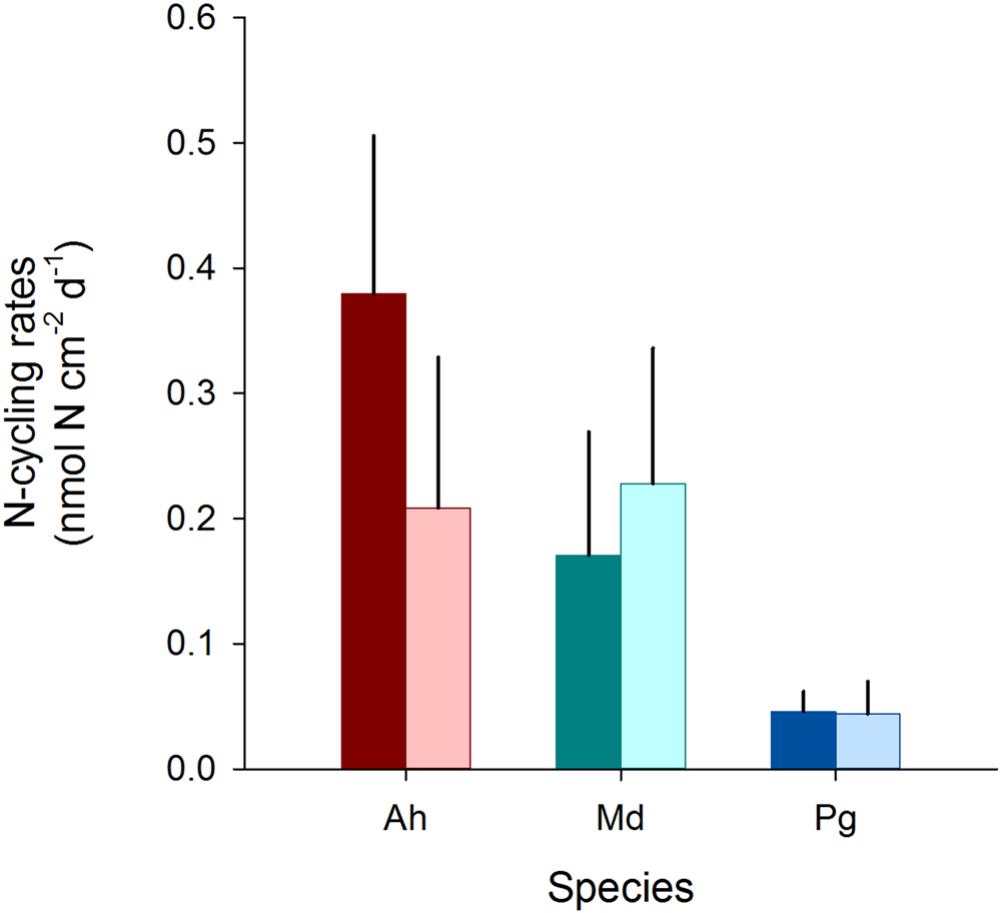
Comparison of denitrification rates and N_2_ fixation rates of three Red Sea coral species. Ah = *Acropora hemprichii*, Md = *Millepora dichotoma*, and Pg = *Pleuractis granulosa*. Bars indicate the mean; error bars indicate upper confidence intervals (+1 SE); dark bars represent denitrification rates; light bars represent N_2_ fixation rates; n = 4 per species.

No significant differences were found between denitrification and N_2_ fixation rates for either species (T-test, *p* > 0.05; Fig. 4).

### Correlation analyses

The biological variable that best explained the denitrification rates was N_2_ fixation (BIOENV, *r* = 0.654, *p* = 0.004). The combination of biological variables that explained denitrification rates best were N_2_ fixation and Symbiodiniaceae cell density (BIOENV, *r* = 0.592, *p* = 0.002). Indeed, 85.6% of the variation in denitrification rates could be explained by N_2_ fixation and Symbiodiniaceae cell density (DistLM).

The biological variable that best explained the N_2_ fixation rates was denitrification (BIOENV, r = 0.651, p = 0.006). The combination of biological variables that explained N_2_ fixation best were denitrification and Symbiodiniaceae cell density (BIOENV, *r* = 0.342, *p* = 0.046). Indeed, 82.9% of the variation in N_2_ fixation rates could be explained by denitrification and Symbiodiniaceae cell density (DistLM).

## Discussion

Despite the importance of N as a key nutrient for the metabolism of symbiotic corals^33^, relatively little is known about the removal of N by microbes in the internal N-cycling of coral holobionts^12,14^. Here, we assessed relative abundances of coral tissue-associated denitrifiers (using relative gene copy numbers of the *nirS* gene as a proxy), as well as denitrification and N_2_ fixation rates on the holobiont level in a comparative taxonomic framework using three common Red Sea corals. Our results suggest that denitrification is an active N-cycling pathway in coral holobionts and may be linked with diazotroph activity and Symbiodiniaceae cell density, the interplay of which may have important implications for coral holobiont nutrient cycling.

It was previously hypothesized that coral associated N-cycling microbes may have a capacity to alleviate nutrient stress via the removal of bioavailable N^14,22,28,29^. While the community structure and phylogenetic diversity of denitrifying microbes have been previously assessed in a soft and a hard coral^29^, we here present the first study to link relative coral tissue-associated abundances of denitrifying prokaryotes with denitrification rates. Our findings highlight that denitrification may play a role in removing bioavailable N from the coral holobiont. The contribution and hence potential functional importance of denitrification may depend on the host species.

In the present study, the highest relative *nirS* gene copy numbers were found in *A. hemprichii*, while lower relative numbers were observed in *M. dichotoma* and *P. granulosa*. The patterns in relative abundance of the *nirS* gene obtained through qPCR were largely reflected in denitrification rates measured using a COBRA assay. As such, these data suggest that relative *nirS* gene abundance may be a suitable proxy of denitrification potential in corals. Small deviations in the patterns observed for both measurements may be potentially explained by a) differences in the community composition of denitrifying microbes; fungi involved in N metabolism may be present in coral holobionts^34^ and likely lack a (homologous) *nirS* gene^35^, by b) the multi-copy nature of the ITS2^36,37^; by relating *nirS* to ITS2, the relative abundances of *nirS* genes could potentially be underestimated, and by c) the potential presence of denitrifying microbes in the coral skeleton^29,38–40^.

The present study identified a positive correlation between denitrification and N_2_ fixation activity across the three Red Sea coral species investigated. As such, denitrification may have the capacity to counterbalance N input from N_2_ fixation in coral holobionts. We here propose that these processes may be indirectly linked by their similar environmental requirements and constraints.

Denitrification as well as N_2_ fixation are anaerobic processes^31,41^. However, in the present study, no relationship was found between O_2_ fluxes and denitrification and N_2_ fixation rates. This strongly suggests that the activity of these anaerobic processes may be spatially or temporally separated from O_2_ evolution in the coral holobiont, or that the involved N-cycling prokaryotes are capable of supporting these processes in the presence of O_2_^42–45^. In addition to anaerobic conditions, most denitrifiers and diazotrophs require organic C as their energy source, i.e. are heterotrophic^9,46–48^. Besides the uptake of organic C from the water column^49^ and heterotrophic feeding by the coral host, the Symbiodiniaceae are the main source of C-rich photosynthates within the holobiont^50^. Notably, the present study showed a positive correlation between denitrification rates with N_2_ fixation combined with Symbiodiniaceae cell density. In addition, a positive correlation was also shown for N_2_ fixation, namely with denitrification combined with Symbiodiniaceae cell density. This suggest that the heterotrophic prokaryotes of both N-cycling pathways may rely partially on Symbiodiniaceae for obtaining organic C for respiration. As such, the correlation of denitrification and N_2_ fixation may be the result of a shared organic C limitation within the holobiont^14^. However, a potential functional relationship between N-cycling prokaryotes and phototrophic Symbiodiniaceae remains yet to be determined.

The observed positive correlation between the two N-cycling pathways, i.e. denitrification and N_2_ fixation, may have important implications for the general understanding of nutrient cycling within coral holobionts, and hence our understanding of coral ecology. In a stable healthy holobiont, N input from N_2_ fixation may be compensated for by N removal via denitrification. As such, the activity of these two processes should be interpreted in relation to each other to understand their overall effect on holobiont N availability, and hence nutrient dynamics.

Environmental stress may directly affect the equilibrium of these processes, as both eutrophication and the increase in sea surface temperatures directly affect N-cycling within the coral holobiont^14^. Increases in inorganic N availability may lead to a reduction of diazotroph activity in coral holobionts due to the so called “ammonia switch-off”^51^, which is evidenced by negative correlations between N availability and N_2_ fixation for both planktonic and benthic diazotrophs^52–54^. Denitrification, on the one hand, may even be stimulated by increased nitrate availability^31^. This hypothesized interplay of denitrification and N_2_ fixation would hence allow coral holobionts to effectively remove excessive N^14^.

Increased sea surface temperatures, on the other hand, may directly stimulate N_2_ fixation^55^. While the environmental drivers for stimulated N_2_ fixation activity are not fully resolved yet, increased diazotrophy may affect holobiont functioning if not compensated for by denitrification activity^10^. However, with increasing water temperature, Symbiodiniaceae may retain more photosynthates for their own metabolism^56^, potentially limiting organic C availability not only for the coral host, but also for heterotrophic microbes, including denitrifiers. Thus, microbial N-cycling may be more important in highly autotrophic coral holobionts, as they rely more on the Symbiodiniaceae for organic C and may be more susceptible to potential nutrient imbalances due to e.g. increased diazotrophic activity^10^. Indeed, the capacity for heterotrophic feeding has been linked to having a lower susceptibility to warming^57–60^ and eutrophication^61^. However, besides the potential ability to remove bioavailable N from the coral holobiont, the role of denitrifiers under (non-)stressful scenarios remains speculative at this point. Thus, future research could focus on several aspects to disentangle a potential role of denitrification in the context of microbial N-cycling within coral holobionts by (a) identifying the spatial niche that denitrifiers occupy and in which abundances; (b) identifying the denitrifiers’ primary energy source(s) under regular and stressed (e.g. eutrophic or warming) conditions; (c) by quantifying and assessing the interplay of denitrification with other N-cycling processes (potentially) ubiquitous in coral holobionts, e.g. N_2_ fixation, nitrification and ANAMMOX, through molecular, physiological and/or isotope analyses; and (d) how the interplay of N-cycling processes in the coral holobiont is altered in global change scenarios.

## Methods

### Sample collection, aquarium facilities, and maintenance

This study was conducted at the King Abdullah University of Science and Technology (KAUST) in Saudi Arabia. Three common coral species were selected (Fig. 2A) and collected at approx. 5 m water depth at the semi-exposed side of the inshore reef Abu Shosha (N22°18′15″, E39°02′56″) located in the Saudi Arabian central Red Sea in November 2017; specifically, the acroporid coral *Acropora hemprichii* (n = 4 colonies), the hydrozoan *Millepora dichotoma* (n = 4 colonies), and the fungiid coral *Pleuractis granulosa* (n = 8 polyps). Coral colonies of the same species were sampled at least 10 m apart to account for genetic diversity. After collection, the corals were transferred to recirculation aquaria filled with reef water on the vessel, and subsequently transported to the wet lab facility of the Coastal and Marine Resources (CMOR) Core Lab at KAUST. The branching corals *A. hemprichii* and *M. dichotoma* were immediately fragmented into two fragments of similar size each. Fragments were distributed into four independent replicate 150 L flow-through tanks, i.e. each tank held two distinct fragments of each branching coral species (see Supplementary Fig. S1). Individual polyps of *P. granulosa* were not fragmented and distributed randomly over the four tanks. Fragments/polyps were left to acclimate for two weeks prior to the start of measurements. All tanks were continuously supplied with sediment-filtered seawater (flow through rate 300 L h^−1^) from inshore reefs located 1.5 km off KAUST with the following parameters: temperature 27 °C, salinity 40 PSU, and dissolved oxygen (O_2_) 6.4 mg O_2_ L^−1^. All fragments were exposed to a photon flux of ~150 µmol m^−2^ s^−1^ ^62^ on a 12:12 h light/dark cycle. Corals were kept in nutrient-rich seawater (nitrate ~3 µM, phosphate ~0.40 µM) to stimulate denitrification response in coral holobionts^63–65^. For each measurement, one fragment/polyp of each species per tank was taken, avoiding sampling fragments that originated from the same colony. This resulted in four fragments/polyps per species from four different colonies for each measurement (see Supplementary Fig. S1).

### DNA extraction and relative quantification of the *nirS* gene via quantitative PCR (qPCR)

Quantitative PCR was used to quantify relative gene copy numbers of the *nirS* gene as a proxy for abundance of denitrifying prokaryotes in the coral tissues, i.e. denitrification potential. To this end, the coral tissue was removed from the skeleton by airblasting with RNase free water and pressurized air using a sterilized airbrush (Agora-Tec GmbH, Schmalkalden, Germany). For *P. granulosa*, tissue was blasted off from both top and bottom surfaces and was pooled subsequently. The resulting tissue slurries were homogenized using an Ultra Turrax (for approx. 20 s) and stored at -20 °C until further processing. Total DNA was extracted from 100 µL of tissue slurry using the Qiagen DNeasy Plant Mini Kit (Qiagen, Germany) according to manufacturer’s instructions. DNA extraction yields were determined using a NanoDrop 2000C spectrophotometer (Thermo Fisher Scientific, Waltham, MA, USA). DNA concentrations were adjusted to 2 ng µL^−1^ and stored at −20 °C until further processing.

qPCR assays were performed in technical triplicates for each coral fragment or polyp. Each assay contained 9 μL reaction mixture and 1 μL DNA template. Reaction mixture contained Platinum SYBR Green qPCR Master Mix (Invitrogen, Carlsbad, CA, United States), 0.2 μL of each primer (10 µM, see below and details of primer assessment in the Supplementary Methods), 0.2 μL of ROX dye and 3.4 μL of RNAse-free water. Negative controls (i.e., reactions consisting of only qPCR reagents and nuclease-free water without any DNA added) were included in the assay in technical triplicates to account for potential laboratory and kit contamination. The relative number of *nirS* gene copies (i.e. relative abundance of denitrifiers) was determined by normalization against the multi copy gene marker ITS2 of Symbiodiniaceae as previously used for the normalization of *nifH* gene copy numbers in a comparative taxonomic coral framework^10^. A total of 18 primers covering all main enzymes in the denitrification pathway (Fig. 1) were tested for this study and yielded ten primer pairs that produced PCR products in the suggested size range (see details of primer assessment in the Supplementary Methods). Temperature gradient PCRs were applied (from 51 °C to 62 °C) to assess the optimal annealing temperature of every primer pair (see details of primer assessment in the Supplementary Methods). Due to substantial differences in amplification performance of primer pairs, we selected a primer pair for *nirS* which encodes for a nitrite reductase containing cytochrome *cd*_1_ as the target gene. For the amplification of *nirS*, the primers cd3aF 5′-GTSAACGTSAAGGARACSGG-3′ and R3cd 5′-GASTTCGGRTGSGTCTTGA-3′ were used^66^. This primer pair was previously found to perform well with DNA from other marine templates, such as coral rock^24^, marine sediments^67^, as well as environmental samples from intertidal zones^68^, and terrestrial ecosystems^47,69,70^. To amplify the ITS2 region of Symbiodiniaceae the primers SYM_VAR_5.8S2 5′-GAATTGCAGAACTCCGTGAACC -3′ and SYM_VAR_REV 5′-CGGGTTCWCTTGTYTGACTTCATGC -3′ were used^71^. The thermal cycling protocol used for the amplification of both target genes was 50 °C for 2 min, 95 °C for 2 min, 50 cycles of 95 °C for 30 s, 51 °C for 1 min, 72 °C for 1 min, and a final 72 °C extension cycle for 2 min. Amplification specificity was determined by adding a dissociation step. All assays were performed on the ABI 7900HT Fast Real-Time PCR System (Applied Biosystems, CA, USA). Standard calibration curves were run simultaneously covering 5 orders of magnitude (10^3^–10^7^ copies of template per assay for the ITS2 and *nirS* gene). The qPCR efficiency (E) of both primer pairs was 84% and 86%, respectively, calculated according to the equation E = [10^(−1/slope)^-1]. Relative fold change of *nirS* gene copies were calculated as 2^(−∆∆Ct)^ against ITS2 Ct values using *P. granulosa* samples as the reference.

Throbäck *et al*.^72^ assessed a range of *nirS* primer pairs and concluded that the primer pair used in the present study (i.e. cd3aF and R3cd) had the largest range and worked best for *nirS* gene assessments. Currently, there are no optimal universal primers for the amplification of the *nirS* gene available^73^. Any quantification of *nirS* abundances is hence biased by the primer pair used and its suitability strongly depends on the phylogenetic diversity of the template. Thus, while the primer combination used here shows a high coverage of 67% of known *nirS* diversity^73^, our results can only provide an approximation of the relative abundance of denitrifying bacteria across samples until more (meta)genomic data for coral-associated denitrifiers are available.

### Denitrification and N_2_ fixation measurements

To measure denitrification and N_2_ fixation rates simultaneously, we incubated corals using a COBRA assay, as described in El-Khaled *et al*. (unpublished). Of note, acetylene inhibits the production of nitrate in the nitrification pathway^74,75^. As nitrate serves as a substrate for denitrification, the inhibition of nitrification may thus result in an underestimation of denitrification rates. To compensate for such effects, nutrient-rich incubation water was used to preclude substrate limitation^63–65^.

Briefly, incubations were conducted in gas-tight 1 L glass chambers, each filled with 800 mL of nutrient-rich sediment-filtered seawater (DIN = ~3 µM, phosphate = ~0.40 µM) and a 200 mL gas headspace. Both incubation water and headspace were enriched with 10% acetylene. Each chamber contained a single *A. hemprichii* or *M. dichotoma* fragment or *P. granulosa* polyp. Incubations of four biological replicates per species were performed (see Supplementary Fig. S1), and three additional chambers without corals served as controls to correct for planktonic background metabolism. During the 24 h incubations, chambers were submersed in a temperature-controlled water bath and constantly stirred (500 rpm) to create a constant water motion and homogenous environment (27 °C, 12:12 h dark/light cycle, photon flux of ~150 µmol m^−2^ s^−1^). Nitrous oxide (N_2_O; as a proxy for denitrification) and ethylene (C_2_H_4_; as a proxy for N_2_ fixation) concentrations were quantified by gas chromatography and helium pulsed discharge detection (Agilent 7890B GC system with HP-Plot/Q column, lower detection limit for both target gases was 0.3 ppm). To facilitate comparisons of both N-cycling processes, N_2_O and C_2_H_4_ production rates were converted into N production using molar ratios of N_2_O:N_2_ = 1 and C_2_H_4_:N_2_ = 3^76^, and multiplying by 2 to convert N_2_ to N, resulting in rates of nmol N cm^−2^ d^−1^. Gas concentrations were normalized to coral surface area, which was calculated using cloud-based 3D models of samples (Autodesk Remake v19.1.1.2)^77,78^.

### Symbiodiniaceae cell density

Tissue slurry for DNA extraction was also used to obtain cell densities of Symbiodiniaceae (see Supplementary Fig. S1). Symbiodiniaceae cell densities were obtained by manual counts of homogenized aliquots of 20 µL, which were diluted 5 times, using a Neubauer-improved hemocytometer on a light microscope with HD camera (Zeiss, Germany). Resulting photographs were analysed using the Cell Counter Notice in ImageJ software (National Institutes of Health, USA). Cell counts for each individual were done in duplicates and subsequently averaged. Finally, cell counts were normalized to coral surface area to obtain cell densities of Symbiodiniaceae for each fragment or polyp.

### O_2_ fluxes

Net photosynthesis (*P*_net_) and dark respiration (*R*_dark_) were assessed from O_2_ evolution/depletion measurements with the same fragments/polyps 2 days prior to using them for denitrification and N_2_ fixation rate measurements. Corals were incubated for 2 h in individual gas-tight 1 L glass chambers, filled with nutrient-rich sediment-filtered seawater (DIN = ~3 µM, phosphate = ~0.40 µM). Each chamber contained a single *A. hemprichii* or *M. dichotoma* fragment or *P. granulosa* polyp. Incubations of four biological replicates per species were performed (see Supplementary Fig. S1), and three additional chambers without corals served as controls to correct for planktonic background metabolism. During the incubations, chambers were submersed in a temperature-controlled water bath (kept at 27 °C) and constantly stirred (500 rpm) to create a continuous water motion and homogenous environment. Light incubations for *P*_net_ were performed under a photon flux of ~150 µmol m^−2^ s^−1^. *R*_dark_ was obtained by incubating in complete darkness. O_2_ concentrations were measured at the start and end of the respective incubation period using an optical oxygen multiprobe (WTW, Germany). O_2_ concentrations at the start of the incubation were subtracted from O_2_ concentrations at the end, corrected for controls and normalized to incubation time and surface area of the corals. *R*_dark_ is presented as a negative rate. Finally, gross photosynthesis (*P*_gross_) was calculated as the difference between *P*_net_ and *R*_dark_ as follows:

$${P}_{{\rm{g}}{\rm{r}}{\rm{o}}{\rm{s}}{\rm{s}}}=({P}_{{\rm{n}}{\rm{e}}{\rm{t}}})\,{\textstyle \text{-}}\,({R}_{{\rm{d}}{\rm{a}}{\rm{r}}{\rm{k}}})$$

### Statistical analyses

Data were analysed using non-parametric permutational multivariate analysis of variance (PERMANOVA) using PRIMER-E version 6 software^79^ with the PERMANOVA+ add on^80^. To test for differences in relative *nirS* gene copy numbers, denitrification rates, N_2_ fixation rates, Symbiodiniaceae cell densities and O_2_ fluxes between species, 1-factorial PERMANOVAs were performed, based on Bray-Curtis similarities of square-root transformed data. Therefore, Type III (partial) sum of squares was used with unrestricted permutation of raw data (999 permutations), and PERMANOVA pairwise tests with parallel Monte Carlo tests were carried out when significant differences were found.

Differences between denitrification and N_2_ fixation rates within each coral species were assessed using SigmaPlot 12.0 (Systat software). T-tests were performed for normally distributed data and Mann-Whitney U tests were performed when data were not normally distributed .

Additionally, to identify the biological variable (single trial variable) and combination of biological variables (multiple trial variables) that “best explains” the denitrification rate pattern of the coral samples, a Biota and/or Environment matching routine (BIOENV) was performed with 999 permutations based on Spearman Rank correlations. A distance-based linear model (DistLM) using a step-wise selection procedure with AICc as a selection criterion was used to calculate the explanatory power of correlating biological variables^79,81^. Finally, the same BIOENV and DistLM routine was performed for N_2_ fixation rates of the coral samples.

As *P. granulosa* consisted of four individual polyps per measurement (no technical replicates originating from the same polyp), data for each variable were averaged and used as a single data point in analyses unless the same individuals were used (see Supplementary Fig. S1). All values are given as mean ± SE.

## Supplementary information

Supplementary Information

## Data Availability

Raw data of the current study are available from the corresponding author on reasonable request.
