# Publisher Correction: Denitrification Aligns with N_2_ Fixation in Red Sea Corals

**DOI:** 10.1038/s41598-020-59353-0

**Published:** 2020-03-06

**Authors:** Arjen Tilstra, Yusuf C. El-Khaled, Florian Roth, Nils Rädecker, Claudia Pogoreutz, Christian R. Voolstra, Christian Wild

**Affiliations:** 1 Marine Ecology Department, Faculty of Biology and Chemistry, University of Bremen, Bremen, 28359 Germany; 2 Red Sea Research Center, King Abdullah University of Science and Technology, Thuwal, 23955-6900, Kingdom of Saudi Arabia; 3 Department of Biology, University of Konstanz, Konstanz, 78464 Germany

Correction to: *Scientific Reports* 10.1038/s41598-019-55408-z, published online 19 December 2019

In the original PDF version of this Article all figures were incorrectly placed in the introduction. This has now been corrected in the PDF; the HTML version of the paper was correct at the time of publication.

