# Supplementary material for: Denitrification Aligns with N2 Fixation in Red Sea Corals
Source: Sci Rep. 2019 Dec 19;9:19460. doi: 10.1038/s41598-019-55408-z (PMC6923481; doi:10.1038/s41598-019-55408-z)
Supplement: Supplementary file 1 — Supplementary Information [file 41598_2019_55408_MOESM1_ESM.pdf]

Supplementary Information

Denitrification aligns with N_2 fixation in Red Sea corals

Arjen Tilstra[1]*, Yusuf C. El-Khaled[1], Florian Roth[2], Nils Rädecker[2], Claudia Pogoreutz[2], Christian R. Voolstra[2,3], Christian Wild[1]

[1]Marine Ecology Department, Faculty of Biology and Chemistry, University of Bremen, Bremen, Germany
[2]Red Sea Research Center, King Abdullah University of Science and Technology, Thuwal, Kingdom of Saudi Arabia
[3]Department of Biology, University of Konstanz, Konstanz, Germany
*tilstra@uni-bremen.de

Supplementary Methods

Primer assessment

Approach

For the purpose of finding suitable primer pairs for the relative quantification of denitrifying prokaryotes, the selected primers were first tested with total DNA from corals previously collected for a comparative assessment of *nifH* gene copy numbers[1] in a temperature gradient PCR (range 51 to 62 °C). If one or more primers gave a single or dominant amplicon of the correct size at a given annealing temperature, the primer(s) and the annealing temperature were selected for testing quantification using quantitative PCR (qPCR). When amplification products of the same samples were similar between conventional PCR and qPCR, the primer(s) and corresponding annealing temperature were further tested on five additional common species of central Red Sea corals. Finally, all additional coral species that revealed a single and/or dominant band of the correct size were tested with qPCR.

Sample collection

This study was conducted at the King Abdullah University of Science and Technology (KAUST) in Saudi Arabia. Two species of Fungiidae (*Pleuractis granulosa* and *Ctenactis echinata*) and two species of Pocilloporidae (*Pocillopora verrucosa* and *Stylophora pistillata*) were collected (N = 3 colonies each) at the inshore reef Inner Fsar (22°13′97.4″N; 39°01′76.0″E) located in the Saudi Arabian central Red Sea in February 2016[1]. Five additional coral species (N = 3 colonies each) were collected at the inshore reef Abu Shosha (22°18′16.3″N; 39°02′57.7″E) located in the Saudi Arabian central Red Sea in September 2017; specifically, two species of Acroporidae (*Acropora hemprichii* and *Acropora pharaonis*), one species of Poritidae (*Porites lutea*), one species of Merulinidae (*Echinopora fruticulosa*) and one hydrozoan (*Millepora dichotoma*). Sailing permits were issued by the Saudi Arabian Coastguard Authority to the sites that included coral collection. Immediately after collection, the coral samples were flash-frozen in liquid nitrogen on board the boat and stored at -80 °C until further processing.

DNA Extraction

For DNA extraction, coral tissues were removed from the skeleton by airblasting with RNase free water using a sterile airbrush (Agora-Tec GmbH, Schmalkalden, Germany). The resulting tissue slurry was collected into sterile 2.0 ml Eppendorf cups, subsequently homogenized and stored at -20 °C until further processing. DNA was extracted from 100 µL of tissue slurry using the Qiagen DNeasy Plant Mini Kit (Qiagen, Germany) according to manufacturer’s instructions. DNA extraction yields were quantified and qualified using a NanoDrop 2000C spectrophotometer (Thermo Fisher Scientific, Waltham, MA, USA) and stored at -20 °C until further processing.

Primer selection and quantitative PCR (qPCR)

A total of 18 primers were selected for this study (Supplementary Table S1), that resulted in 10

primer pairs selected for further testing (Supplementary Table S2 and S3). Primer selection was done by examining the scientific literature for primers that have been used to assess denitrifying microbes preferably performed associated with coral reef organisms or substrates. If unavailable, denitrification primers that have been used on samples from other marine ecosystems were selected. A minimum of two primer pairs targeting functional genes encoding for each enzyme present in the denitrification pathway were selected, i.e. the *narG* gene encoding for nitrate reductase, the *nirK* and *nirS* genes encoding for nitrite reductase, the *norB* gene for nitric oxide reduction and the *nosZ* gene encoding for nitrous oxide reduction (Supplementary Table S1).

A temperature gradient PCR was applied (from 51 °C to 62 °C) to assess the optimal annealing temperature of every primer pair. All PCRs were run in duplicates containing 10 µL of Qiagen Multiplex mix (from the Qiagen Multiplex PCR Kit), 0.5 µM of each primer (10 µM), 1 µL of DNA template and PCR water to adjust the total reaction volume to 20 µL. The thermal cycler protocol was 94 °C for 15 min, followed by 50 cycles of 94 °C for 30 s, 51 to 62 °C for 1 min followed by 72 °C for 1 min, with a final extension at 72 °C for 10 min. The amplification products were visually analysed using 1 % agarose gel electrophoresis in 1x TAE buffer.

Quantitative PCR (qPCR) was used to test primers that gave a single or dominant amplicon of the correct size with conventional PCR. qPCR assays were performed in triplicates for each coral replicate. Each assay contained 9 µL reaction mixture and 1 µL DNA template. Reaction mixture contained Platinum SYBR Green qPCR Master Mix (Invitrogen, Carlsbad, CA, United States), 0.2 µL of each primer (10 µM), 0.2 µL of ROX dye and 3.4 µL of RNAse-free water. The thermal cycling protocol was 50 °C for 2 min, 95 °C for 2 min, 50 cycles of 95 °C for 30 s, "optimal annealing temperature" for 1 min, 72 °C for 1 min and a 72 °C extension cycle for 2 min. Amplification specificity was determined by adding a dissociation step. All assays were performed on the ABI 7900HT Fast Real-Time PCR System (Applied Biosystems, CA, USA). The amplification products were visually analysed using 1 % agarose gel electrophoresis in 1x TAE buffer.

Results

In the first batch of tested corals, amplicons of the correct size (425 bp) were found in *S. pistillata* and *P. granulosa* with the primer pair cd3aF/R3cd (Supplementary Fig. S2 and Supplementary Table S2). Clearest amplicons (as qualified with gel electrophoresis) were found with an annealing temperature of 51 °C (Supplementary Fig. S2 and Supplementary Table S2). Results of subsequent qPCRs mirrored results obtained from conventional PCRs. For the second batch of corals the primer pair cd3aF/R3cd was used with an annealing temperature of 51 °C. In addition to *S. pistillata* and *P. granulosa*, amplicons of the correct size were found in *A. hemprichii* and *M. dichotoma* (Supplementary Fig. S3 and Supplementary Table S3). Amplifiation products of subsequent qPCRs mirrored products obtained from conventional PCRs for each sample.

Primer assessment

Here, we studied the performance of 10 primer pairs targeting five functional genes of the denitrification pathway and encoding for the involved main enzymes. Specifically, the selected primer pairs targeted the denitrification gene clusters *nar*, *nir*, *nor* and *nos*. Based on primer performance for DNA isolated from coral, we selected the primer pair cd3af/R3cd which targets the *nirS* gene encoding for the cytochrome cd_1 nitrite reductase. This primer pair was the only pair to give a dominant band of the correct size for several tested corals. Of note, this primer pair was previously found to perform well with DNA from other marine templates, such as coral rock[2], marine sediments[3], as well as environmental samples from intertidal zones[4], and terrestrial ecosystems[5–7], highlighting its broad coverage.

References

1. Pogoreutz, C. *et al.* Nitrogen fixation aligns with *nifH* abundance and expression in two coral trophic functional groups. *Front. Microbiol.* **8**, 1187 (2017).
2. Yuen, Y. S., Yamazaki, S. S., Nakamura, T., Tokuda, G. & Yamasaki, H. Effects of live rock on the reef-building coral *Acropora digitifera* cultured with high levels of nitrogenous compounds. *Aquac. Eng.* **41**, 35–43 (2009).
3. Nakano, M., Shimizu, Y., Okumura, H., Sugahara, I. & Maeda, H. Construction of a consortium comprising ammonia-oxidizing bacteria and denitrifying bacteria isolated from marine sediment. *Biocontrol Sci.* **13**, 73–89 (2008).
4. Dini-Andreote, F., Brossi, M. J. L., van Elsas, J. D. & Salles, J. F. Reconstructing the genetic potential of the microbially-mediated nitrogen cycle in a salt marsh ecosystem. *Front. Microbiol.* **7**, 902 (2016).
5. Jung, J. *et al.* Change in gene abundance in the nitrogen biogeochemical cycle with temperature and nitrogen addition in Antarctic soils. *Res. Microbiol.* **162**, 1018–1026 (2011).
6. Jung, J., Yeom, J., Han, J., Kim, J. & Park, W. Seasonal changes in nitrogen-cycle gene abundances and in bacterial communities in acidic forest soils. *J. Microbiol.* **50**, 365–373 (2012).
7. Chen, S. *et al.* Organic carbon availability limiting microbial denitrification in the deep vadose zone. *Environ. Microbiol.* **20**, 980–992 (2018).
8. Gregory, L. G., Karakas-Sen, A., Richardson, D. J. & Spiro, S. Detection of genes for membrane-bound nitrate reductase in nitrate-respiring bacteria and in community DNA. *FEMS Microbiol. Lett.* **183**, 275–279 (2000).
9. Bru, D., Sarr, A. & Philippot, L. Relative abundances of proteobacterial membrane-bound and periplasmic nitrate reductases in selected environments. *Appl. Environ. Microbiol.* **73**, 5971–5974 (2007).
10. Braker, G., Fesefeldt, A. & Witzel, K.-P. Development of PCR primer systems for amplification of nitrite reductase genes (*nirK* and *nirS*) to detect denitrifying bacteria in environmental samples. *Appl. Environ. Microbiol.* **64**, 3769–3775 (1998).
11. Zhang, X., He, L., Zhang, F., Sun, W. & Li, Z. The different potential of sponge bacterial symbionts in N_2 release indicated by the phylogenetic diversity and abundance analyses of denitrification genes, *nirK* and *nosZ*. *PLoS One* **8**, 18–20 (2013).
12. Michotey, V., Méjean, V. & Bonin, P. Comparison of methods for quantification of cytochrome cd1-denitrifying bacteria in environmental marine samples. *Appl. Environ. Microbiol.* **66**, 1564–1571 (2000).

13. Braker, G. & Tiedje, J. M. Nitric oxide reductase (*norB*) genes from pure cultures and environmental samples nitric oxide reductase (*norB*) genes from pure cultures and environmental samples. *Appl. Environ. Microbiol.* **69**, 3476–3483 (2003).
14. Kloos, K., Mergel, A., Rösch, C. & Bothe, H. Denitrification within the genus *Azospirillum* and other associative bacteria. *Aust. J. Plant Physiol.* **28**, 991–998 (2001).
15. Throbäck, I. N., Enwall, K., Jarvis, Å. & Hallin, S. Reassessing PCR primers targeting *nirS*, *nirK* and *nosZ* genes for community surveys of denitrifying bacteria with DGGE. *FEMS Microbiol. Ecol.* **49**, 401–417 (2004).

Supplementary Tables

Supplementary Table S1. Selected primers used for amplification of denitrification genes

Enzyme	Target gene	Primer[a]	Nucleotide sequence (5’ → 3’)[b]	Reference
Nitrate reductase	*narG*	narGW9F	MGNGGNTGYCCNMGNGGNGC	Gregory et al.[8]
		narGT38R	ACRTCNGTYTGYTCNCCCCA	
		narG-F	TCGCCSATYCCGGCSATGTC	Bru et al.[9]
		narG-R	GAGTTGTACCAGTCRGCSGAYTCSG	
Nitrite reductase	*nirK*	nirK1F	GGMATGGTKCCSTGGCA	Braker et al.[10]
		nirK5R	GCCTCGATCAGRTTRTGG	
		nirK127R	CCTGCTCACCGACATAATAGA	Zhang et al.[11]
Nitrite reductase	*nirS*	nirS1F	CCTAYTGGCCGCCRCART	Braker et al.[10]
		nirS6R	CGTTGAACTTRCCGGT	
		cd3aF	GTSAACGTSAAGGARACSGG	Michotey et al.[12]
		R3cd	GASTTCGGRTGSGTCTTGA	
Nitric oxide reductase	*norB*	qnorB2F	GGNCAYCARGGNTAYGA	Braker and Tiedje[13]
		qnorB5R	ACCCANAGRTGNACNACCCACCA	
		cnorB2F	GACAAGNNNTACTGGTGGT	
		cnorB6R	GAANCCCCANACNCCNGC	
Nitrous oxide reductase	*nosZ*	nosZ-F	CGYTGTTCMTCGACAGCCAG	Kloos et al.[14]
		nosZ-R	CGSACCTTSTTGCCSTYGCG	
		nosZ1622R	CGCRASGGCAASAAGGTSCG	Throbäck et al.[15]

[a] Forward and reverse primers are indicated by the letters F and R, respectively.
[b] K = G or T; M = A or C; N = A, C, G or T; R = A or G; S = G or C; Y = C or T

Supplementary Table S2. Amplification results[a] of PCR using a range of primers for the denitrification pathway on four Red Sea corals[b] at different annealing temperatures

Primer pair	Coral species	51.0 °C	51.9 °C	54.1 °C	57.0 °C	59.8 °C	62.0 °C
narGW9F/narGT38R	Pg	–	–	–	–	–	–
	Ce	–	–	–	–	–	–
	Pv	–	–	–	–	–	–
	Sp	–	–	–	–	–	–
narG-F/narG-R	Pg	–	–	–	–	–	–
	Ce	nsa	nsa	–	–	–	–
	Pv	–	–	–	–	–	–
	Sp	nsa	nsa	–	–	–	–
nirK1F/nirK5R	Pg	nsa	nsa	nsa	nsa	–	–
	Ce	nsa	nsa	nsa	nsa	nsa	nsa
	Pv	nsa	nsa	nsa	–	–	nsa
	Sp	nsa	nsa	nsa	nsa	nsa	nsa
nirK1F/nirK127R	Pg	nsa	nsa	–	–	–	–
	Ce	nsa	nsa	nsa	nsa	nsa	nsa
	Pv	nsa	nsa	nsa	nsa	–	nsa
	Sp	nsa	nsa	nsa	nsa	nsa	nsa
nirS1F/nirS6R	Pg	–	–	–	–	–	–
	Ce	nsa	nsa	nsa	nsa	nsa	–
	Pv	nsa	nsa	nsa	nsa	nsa	–
	Sp	nsa	nsa	nsa	nsa	nsa	–
cd3aF/R3cd	Pg	+	+	+	+	+	–
	Ce	nsa	nsa	–	–	–	–
	Pv	nsa	nsa	–	–	–	–
	Sp	+	+	+	+	+	–
qnorB2F/qnorB5R	Pg	–	–	–	–	–	–
	Ce	–	–	–	–	–	–
	Pv	–	–	–	–	–	–
	Sp	–	–	–	–	–	–
cnorB2F/cnorB6R	Pg	–	–	–	–	–	–
	Ce	nsa	nsa	–	–	–	–
	Pv	nsa	nsa	–	–	–	–
	Sp	–	–	–	–	–	–
nosZ-F/nosZ-R	Pg	nsa	nsa	nsa	nsa	nsa	nsa
	Ce	nsa	nsa	nsa	nsa	nsa	–
	Pv	nsa	nsa	nsa	nsa	nsa	nsa
	Sp	nsa	nsa	–	–	–	–
nosZ-F/nosZ1622R	Pg	nsa	nsa	–	–	–	–
	Ce	nsa	nsa	nsa	nsa	nsa	–
	Pv	nsa	nsa	nsa	nsa	nsa	–
	Sp	nsa	nsa	–	–	–	–

[a] Symbols: Amplicon of the correct size (+); no amplicon (–); multiple amplicons or single amplicon of the wrong size, i.e. no specific amplification (nsa)

[b] Corals are abbreviated as follows: *Pleuractis granulosa* (Pg); *Ctenactis echinata* (Ce); *Pocillopora verrucosa* (Pv); *Stylophora pistillata* (Sp)

Supplementary Table S3. Amplification results[a] of PCR using a range of primers for the denitrification pathway on nine Red Sea corals[b]

Primer pair[c]	Amplicon length[d]	Pg	Ce	Pv	Sp	Ah	Ap	Pl	Ef	Md	C
narGW9F/narGT38R	500 bp	–	–	–	–						–
narG-F/narG-R	173 bp	–	nsa	–	nsa						–
nirK1F/nirK5R	514 – 515 bp	nsa	nsa	nsa	nsa						–
nirK1F/nirK127R	127 bp	nsa	nsa	nsa	nsa						–
nirS1F/nirS6R	890 bp	–	nsa	nsa	nsa						–
cd3aF/R3cd	425 bp	+	nsa	nsa	+	+	nsa	–	nsa	+	–
qnorB2F/qnorB5R	224 – 262 bp	–	–	–	–						–
cnorB2F/cnorB6R	389 bp	–	nsa	nsa	–						–
nosZ-F/nosZ-R	453 bp	nsa	nsa	nsa	nsa						–
nosZ-F/nosZ1622R	415 – 453 bp	nsa	nsa	nsa	nsa						–

[a] Symbols: Amplicon of the correct size (+); no amplicon (–); multiple amplicons or single amplicon of the wrong size, i.e. no specific amplification (nsa), empty cells indicate that the assessment was not performed

[b] Corals are abbreviated as follows: *Pleuractis granulosa* (Pg); *Ctenactis echinata* (Ce); *Pocillopora verrucosa* (Pv); *Stylophora pistillata* (Sp); *Acropora hemprichii* (Ah); *Acropora pharaonis* (Ap); *Porites lutea* (Pl); *Echinopora fruticulosa* (Ef); *Millepora dichotoma* (Md); Negative control (C).

[c] Annealing temperature of 51 °C for every primer pair

[d] Basepairs (bp)

Supplementary Figures

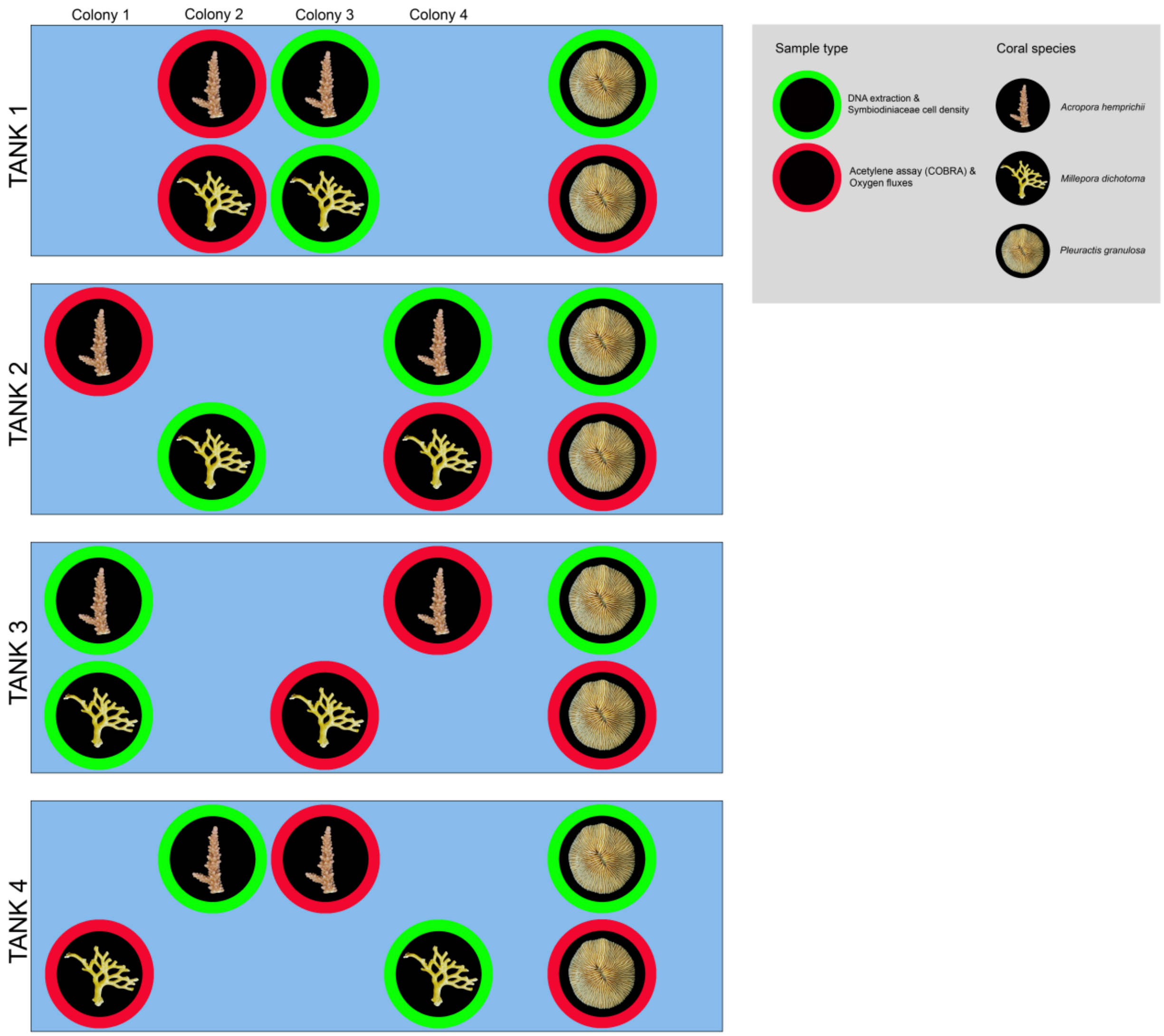

Supplementary Figure S1. Overview of sampling scheme of fragmented coral colonies, i.e. 4 colonies of *A. hemprichii*, 4 colonies of *M. dichotoma* and 8 individual polyps of *P. granulosa*, which were divided over 4 independent replicate tanks. The outline colour denotes the type of analysis that the sampled fragments were used for.

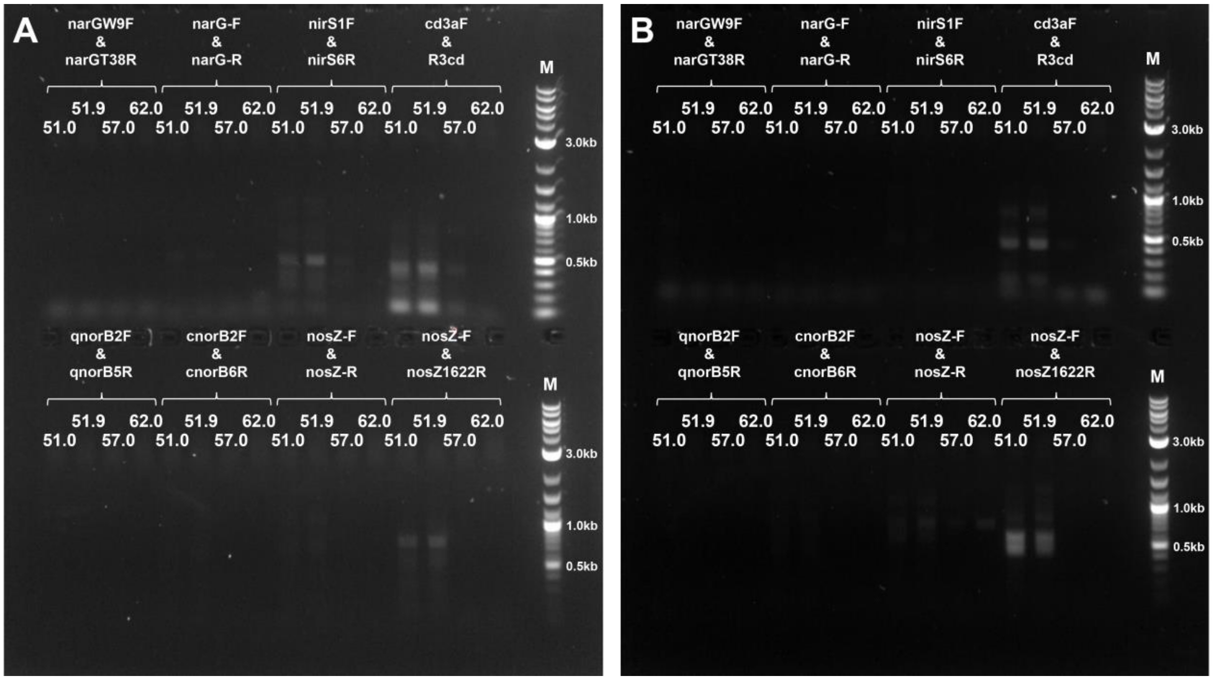

Supplementary Figure S2. PCR products from eight different primer pairs at four different annealing temperatures for (A) *Stylophora pistillata* and (B) *Pleuractis granulosa*. Annealing temperatures are 51.0, 51.9, 57.0 and 62.0 °C. M = Marker (2-log DNA ladder). Uncropped gel images originate from two separate gels.

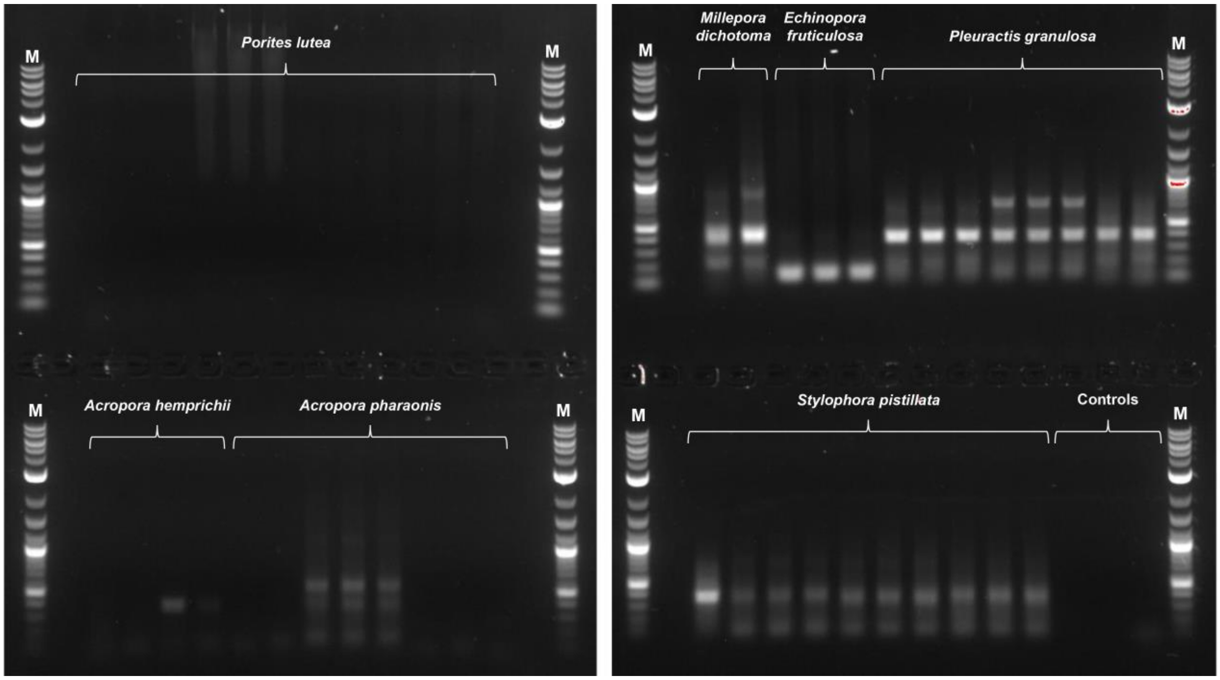

Supplementary Figure S3. PCR products using the primer pair cd3aF/R3cd at the optimal annealing temperature of 51 °C using 7 different Red Sea corals. M = Marker (2-log DNA ladder). Uncropped gel images originate from two separate gels.
